# A Novel Mechanism of Formaldehyde Neurotoxicity: Inhibition of Hydrogen Sulfide Generation by Promoting Overproduction of Nitric Oxide

**DOI:** 10.1371/journal.pone.0054829

**Published:** 2013-01-24

**Authors:** Xiao-Qing Tang, Heng-Rong Fang, Cheng-Fang Zhou, Yuan-Yuan Zhuang, Ping Zhang, Hong-Feng Gu, Bi Hu

**Affiliations:** 1 Department of Physiology, Medical College, University of South China, Hengyang, Hunan, P. R. China; 2 Department of Pharmacy, Hengyang Central Hospital, Hengyang, Hunan, P. R. China; 3 Department of Neurology, Nanhua Affiliated Hospital, University of South China, Hengyang, Hunan, P. R. China; University of Louisville, United States of America

## Abstract

**Background:**

Formaldehyde (FA) induces neurotoxicity by overproduction of intracellular reactive oxygen species (ROS). Increasing studies have shown that hydrogen sulfide (H_2_S), an endogenous gastransmitter, protects nerve cells against oxidative stress by its antioxidant effect. It has been shown that overproduction of nitric oxide (NO) inhibits the activity of cystathionine-beta-synthase (CBS), the predominant H_2_S-generating enzyme in the central nervous system.

**Objective:**

We hypothesize that FA-caused neurotoxicity involves the deficiency of this endogenous protective antioxidant gas, which results from excessive generation of NO. The aim of this study is to evaluate whether FA disturbs H_2_S synthesis in PC12 cells, and whether this disturbance is associated with overproduction of NO.

**Principal Findings:**

We showed that exposure of PC12 cells to FA causes reduction of viability, inhibition of CBS expression, decrease of endogenous H_2_S production, and NO production. CBS silencing deteriorates FA-induced decreases in endogenous H_2_S generation, neurotoxicity, and intracellular ROS accumulation in PC12 cells; while ADMA, a specific inhibitor of NOS significantly attenuates FA-induced decreases in endogenous H_2_S generation, neurotoxicity, and intracellular ROS accumulation in PC12 cells.

**Conclusion/Significance:**

Our data indicate that FA induces neurotoxicity by inhibiting the generation of H_2_S through excess of NO and suggest that strategies to manipulate endogenous H_2_S could open a suitable novel therapeutic avenue for FA-induced neurotoxicity.

## Introduction

Formaldehyde (FA), a pungent, highly flammable and colorless gas, is a well-known indoor and outdoor pollutant. Everyone is exposed to FA from many sources, including exhaust gas, cigarette smoke, household products, and many other medical and industrial products. FA has many detrimental effects on various tissues including skin, eye, gonads, the gastrointestinal system and the respiratory tract [1]. Recently, the neurotoxic effects of FA in the human health have attracted extensive studies. Epidemiological data showed that neurocognitive and neurobehavioral impairment occur in histology technicians and workers exposed to high levels of FA over a long time [2], [3]. The neurotoxic effects of FA have been confirmed in several experimental models. It has been shown that FA induces neurotoxic effects in the cultured cortical neurons and PC12 cells in vitro [4]–[6]. The neurotoxicity of FA has also been confirmed by animal studies that exposure of rats to FA causes various morphological changes in the brain [7] and damages the prefrontal cortex including the hippocampus [8], [9] and that Inhaled FA leads to learning and memory disorders in rats and mice [10]–[12]. Moreover, abundant evidence confirms that the increased endogenous FA levels by upregulation of semicarbazide-sensitive amine oxidase (SSAO), one of the enzymes in the pathway producing FA [13], and deficiency of aldehyde dehydrogenase class 2 (ALDH-2), one of the enzymes that degrade FA [14], contribute to the pathology of Alzheimer’s disease [15]–[17]. Although the wide distribution of FA in the environment and its serious threats to brain, the detailed mechanisms underlying the neurotoxicity of FA have not been fully elucidated.

Increasing evidence demonstrated that oxidative damage is one of the most critical effects of FA exposure [8]–[10], [18], [19]. Oxidative stress is the process of cellular injury caused by excessive levels of ROS, resulting from an imbalance between pro-oxidant and antioxidant systems. When ROS formation is unbalanced in proportion to protective antioxidants, the excess ROS cause toxic effects and ultimately lead to cell death [20], [21]. Hydrogen sulfide (H_2_S) has been reported as an endogenous antioxidant gas [22]. H_2_S protects primary rat cortical neurons from oxidative insult by stimulating GSH synthesis [23] and protects SHSY-5Y cells from oxidative damage by scavenging peroxynitrite (ONOO^ ¯^) [24] and hypochlorous acid (HOCl) [25]. It was recently demonstrated that H_2_S protects PC12 and SH-SY5Y cells against oxidative stress induced by MPP^+^ [26], Aβ_25–35_ [27], homocysteine [28], 6-OHDA [29] and CoCl_2_ [30]. Taken together, these findings provide evidence that H_2_S has potential therapeutic value for oxidative stress-induced neural damage. Disturbed H_2_S synthesis has been shown to contribute to 1-methy-4-phenylpyridinium ion (MPP^+^)- and homocystene-induced oxidative stress and neurotoxicity [31], [32]. This raises questions whether FA disturbs H_2_S synthesis and whether FA-caused neurotoxicity involves the imbalance of proportion to this endogenous protective antioxidant gas.

Endogenous H_2_S is from cysteine by two pyridoxal-5-phosphate- dependent enzymes, namely cystathionine β-synthase (CBS) [33] and cystathionine γ-lyase (CSE) [34]. CBS is mainly expressed in the nervous system [35], whereas CSE appears to be predominant in the cardiovascular system [34]. It has been proved that inhaled FA increases the levels of nitric oxide (NO) in the rat cerebellum [36] as well as that NO binds to CBS and inhibits the activity of CBS [37], [38]. Therefore, in this study, we further elucidate the role of NO in the disturbance of H_2_S synthesis caused by FA.

## Materials and Methods

### 1. Materials

Hoechst 33258, propidium iodide (PI), RNase, rhodamine 123 (Rh123), 2′,7′-dichlorfluorescein-diacetate (DCFH-DA), asymmetric dimethylarginine (ADMA, dissolved in dimethyl sulfoxide) and formaldehyde [dissolved in Phosphate Buffered Saline(PBS)] were purchased from Sigma Chemical CO (st.Louis, MO, USA). Cell counting kit-8 (CCK-8) was supplied by Dojindo Molecular Technologies, Inc. (Rockvile, MD, USA). Specific monoclonal antibodies to CBS and 3-mercaptopyruvate sulfur transferase (3-MST) were obtained from Santa Cruz Biotechnology, Inc. (Santa Cruz, CA, USA). The short hairpin RNA (shRNA) targeting rat CBS gene (shRNA CBS) and the scrambled shRNA control (shRNA control) were obtained from OriGene Technology Inc (Rockville, MD, USA). X-tremeGENE HP DNA Transfection reagent was supplied by Roche Diagnostics (Indianapoli, IN, USA). Enzyme-linked immunosorbent assay (ELISA) Kits for endothelial nitic oxide synthase (eNOS), neuronal NOS (nNOS), and inducible NOS (iNOS) were from USCN Life Science Inc (Wuhan, Hubei, China). NO kit was purchased from Nanjing Jiancheng Bioengineering Institute (Nanjing, Jiangsu, China). RPMI-1640 medium, horse serum and fetal bovine serum were supplied by Gibico BRL (Ground Island, NY, USA).

### 2. Cell Culture

PC12 cells, originally derived from a transplantable rat pheochromocytoma, were supplied from Sun Yat-sen University Experimental Animal Center (Guangzhou, China), and were maintained on tissue culture plastic in RPMI-1640 medium supplemented with 10% heat-inactivated horse serum and 5% fetal bovine serum (FBS) at 37°C under an atmosphere of 5% CO_2_ and 95% air. The culture media was changed three times per week.

### 3. RNA Interference (RNAi)

We have found that PC12 cells generate H_2_S by cystathionine-β-synthetase (CBS), not by cystathionine-γ-lyase (CSE) [31]. Thus, knockdown of CBS in PC12 cells was accomplished using shRNA CBS (the shRNA target sequences is TGGATAGGTGGTTCAA GAGCAATGATGAC). PC12 cells were plated in 6- or 96-well plates overnight to form 50–70% confluent monolayers. The contents of 2 µg shRNA CBS and 6 µl X-treme GENE HP DNA Transfection Reagent were diluted in serum-free DMEM for final volume of 200 µl, gently mixed, and incubated for 20 min at room temperature. The shRNA CBS and the X-tremeGENE HP DNA transfection reagent complex were added to cells in complete culture medium (200 µl/well at 6-well plates or 10 µl/well at 96-well plates) and incubated in 37°C under an atmosphere of 5% CO_2_ and 95% air for 6 h. A scrambled shRNA was used to transfect PC12 cells as a control.

### 4. Determination of Cell Viability

The viability of PC12 cells was determined by CCK-8 assay. PC12 cells were cultured in 96-well plates at 37°C under an atmosphere of 5% CO_2_ and 95% air. When the cells were about 70% fusion, indicated conditioned-mediums were administered. At the end of treatments, 5 µl CCK-8 solutions were added into each well and then the plates were incubated for further 3 h in the incubator. Absorbance at 450 nm was measured with a microplate reader (Molecular Devices, Sunnyvale, CA, USA). Means of five wells optical density (OD) in the indicated groups were used to calculate the percentage of cell viability according to the formula below: cell viability (%) = OD treatment group/OD control group×100%. The experiment was repeated three times.

### 5. Nuclear Staining for Assessment of Apoptosis

Chromosomal condensation and morphological changes in the nucleus of PC12 cells were observed using the chromatin dye Hoechst 33258. The PC12 cells were fixed with 4% paraformaldehyde in 0.1 M phosphate buffered saline (PBS) for 10 min. After three rinses with PBS, the cells were stained with 5 mg/L Hoechst 33258 for 10 min. Slides were rinsed briefly with PBS, air dried, and then mounted in an anti-fluorescein fading medium (Perma Fluor, Immunon, PA, USA). Slides were visualized under a fluorescent microscope (BX50-FLA, Olympus, Tokyo, Japan). Viable cells displayed normal nuclear size and uniform fluorescence, whereas apoptotic cells showed condensed nuclei or nuclear condensations.

### 6. Flow Cytometry Analysis of Apoptosis

Treated PC12 cells were digested with trypsin (2.5 g/L) and centrifuged at 250 *g* for 10 min and the supernatant removed. Cells were washed twice with PBS and fixed with 70% ethanol. Cells were then centrifuged at 250 *g* for 10 min, washed in PBS twice and adjusted to a concentration of 1×10^6^ cells/ml. To a 0.5 ml cell sample, 0.5 mL RNase (1 mg/mL in PBS) was added. After gentle mixing with PI (at a final concentration of 50 mg/L), mixed cells were filtered and incubated in the dark at 4°C for 30 min before flow cytometric (FCM, Beckman-Coulter, Miami, FL, USA) analysis. In the DNA histogram, the amplitude of the sub-G1 DNA peak represents the number of apoptotic cells.

### 7. Measurement of Intracellular ROS Generation

Intracellular ROS were determined by oxidative conversion of cell permeable 2′,7′-dichlorfluorescein-diacetate (DCFH-DA) to fluorescent 2′,7′-dichlorfluorescein (DCF) [39], [40]. At the end of treatments, DCFH-DA (2.5 µM) was added to the cells and incubated in 37°C for 20 min. After washed twice with PBS, the fluorescence of DCF in cells was measured over the whole field of vision using a fluorescent microscope connected to an imaging system (BX50-FLA, Olympus, Tokyo, Japan).

### 8. Measurement of Nitric Oxide in Culture Supernatant

The level of NO in the culture medium was determined using a NO kit. The method involved measuring the levels of NO metabolites (nitrite and nitrate), which are more stable than NO. We thus estimated the level of NO in the sample by determining total nitrate and nitrite concentrations. Briefly, cell culture medium was incubated with 250 mU/mL nitrate reductase and 100 µM NADPH for 0.5 h at 37°C to reduce nitrate to nitrite. They were then mixed (1∶1) with Griess reagent (1% sulfanilamide +0.1% naphthylethylenediamine dihydrochloride in 2.5% H_3_PO_4_) and incubated for 10 min at room temperature. The absorbance of NO_2_^–^ was read at 540 nm using a microplate reader (Molecular Device, Sunnyvale, CA, USA). Nitrite was calculated against a calibration curve of NaNO_2_ and the result was standardized to the protein level (determined using the Bradford assay).

### 9. Enzyme-Linked Immunosorbent Assay for eNOS, nNOS, and iNOS

PC12 cells were collected and washed twice with PBS, and then homogenized in 50 mM ice-cold potassium phosphate buffer (pH 6.8). The levels of NOS isoforms in the homogenate of PC12 cells were measured using ELISA kits according to manufacturer instructions. Briey, a 96-well microplate was coated with an antibody specific for rat eNOS, nNOS, or iNOS, respectively. Sample (100 µl) was added in duplicate to the microplates and incubated for 2 h and then the liquid of each well was removed. Subsequently, 100 µl of biotinylated anti-eNOS, -nNOS, or –iNOS antibody solution was added and incubated for 45 min and then washed. Streptavidin-horseradish peroxidase conjugate solution (100 µl) was added and incubated for 45 min and washed. Finally, 100 µl of substrate solution was added and incubated in the dark for 15 min. The reaction was stopped with HCl and read at 450 nm using an ELISA plate reader. Standard curves were made with rat eNOS, nNOS, or iNOS as a standard.

### 10. Measurement of H_2_S in Cell Culture Supernatant

The basis of the assay is that H_2_S produced in the incubate reacts with zinc acetate to form zinc sulphide which then dissolves in a hydrochloride acid solution of *N*,*N*-dimethyl-*p*-phenylenediamine sulphate (NNDPD) yielding, in the presence of ferric chloride, methylene blue, which is quantitated spectrophotometrically.

Cell culture supernatant (310 µl) were mixed with trichloroacetic acid (20% w/v, 60 µl), zinc acetate (2% w/v, 30 µl), NNDPD (20 mM; 40 µl) in 7.2 M HCl and FeCl_3_ (30 mM; 30 µl) in 1.2 M HCl. The absorbance of the resulting solution (670 nm) was measured 15 min thereafter by spectrophotometry. H_2_S was calculated against a calibration curve of NaHS.

### 11. Assay of H_2_S Synthesizing Activity

PC12 cells were collected and washed twice with PBS, and then homogenized in 50 mM ice-cold potassium phosphate buffer (pH 6.8). In the absence or presence of L-cysteine (20 µl, 10 mM), tissue homogenate (11% w/v, 430 µl) was added to a reaction mixture, which contained 100 mM potassium phosphate buffer (pH 7.4), pyridoxyal 5′-phosphate (20 µl, 2 mM), saline (30 µl). The reaction was performed in tightly stoppered cryovial test tubes and initiated by transferring the tubes from ice to a shaking water bath at 37°C. After incubation for 30 min, 1% w/v zinc acetate (250 µl) was added to trap evolved H_2_S followed by 10% v/v trichloroacetic acid (250 µl) to denature the protein and stop the reaction. Subsequently, NNDPD (20 µM; 133 µl) in 7.2 M HCl was added, immediately followed by FeCl_3_ (30 µM; 133 µl) in 1.2 M HCl. The absorbance of the resulting solution at 670 nm was measured by spectrophotometry. The H_2_S concentration was calculated against a calibration curve of NaHS. H_2_S synthesizing activity was obtained as the difference in the generation of H_2_S from reaction samples in the absence and presence of L-cysteine and calculated as H_2_S formed from g protein (determined using the Bradford assay) per minute. For every experiment, H_2_S synthesizing activity of PC12 cells exposed to normal conditioned medium was defined as 100% and H_2_S synthesizing activity under other conditions was expressed as a percentage of control.

### 12. Western Blot Analysis for Expressions of CBS and 3-MST

SDS–polyacrylamide gel electrophoresis (PAGE) was carried out on 5% stacking and 12% resolving gel with low range molecular weight standards (Solarbio, China). Equal amounts of protein were loaded in each lane with loading buffer (Beyotime, China) containing 0.1 M Tris (pH6.8), 20% glycerol, 10% mercaptoethanol, 4% SDS and 0.2% Bromophenol Blue. Samples were heated at 100°C for 5 min before loading. Following electrophoresis, the proteins were transferred into a PVDF transfer membrane (Solarbio, China). After this, the membranes were blocked with TBST (50 mM Tris–HCl, pH 7.4, 0.15 M NaCl, 0.1% Tween-20) containing 5% BSA (Sigma, USA) for 2 h. Following this, the membranes were incubated with the primary antibodies diluted 1∶1000 at 4°C over night. After three washes with TBST, the membranes were incubated with anti-rabbit IgG labeled with horseradish peroxidase (Zsbio, China) diluted at 1∶1000 at room temperature for 2 h. The membranes were washed again and developed with an enhanced chemiluminescence system (ECL, Zsbio, China) followed by apposition of the membranes with autoradiographic films (Kodak, China). The integrated optical density for the protein band was calculated by ImageJ 1.4 6i software.

### 13. Statistical Analysis

Data are expressed as mean ± SEM. The significance of inter-group differences was evaluated by one-way analyses of variance (ANOVA) and Least-significant difference (LSD) test. Differences were considered significant at *P*<0.05.

## Results

### 1. Formaldehyde Attenuates the Cell Viability and the Production of H_2_S in PC12 Cells

**Figure 1:**
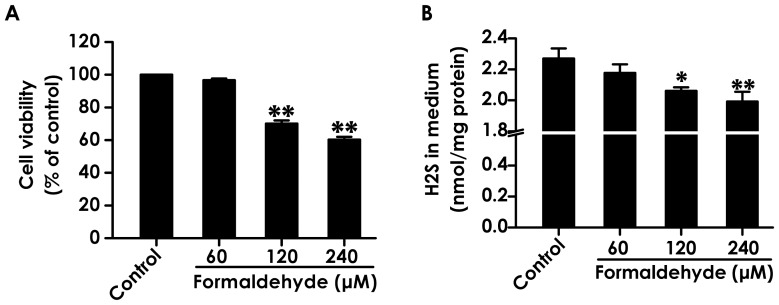
Effects of formaldehyde on the cell viability and endogenous H_2_S production in PC12 cells. The cell viability (A) was determined by CCK-8 assay and the content of H_2_S in cell culture supernatant (B) was tested by the N,Ndimethyl-p-phenylenediamine sulphate (NNDPD) method as described in “Materials and Methods” section after treatment of PC12 cells with formaldehyde (60, 120, or 240 µmol/L) for 24 h. Values are expressed as the mean ± SEM of three independent experiments. ^*^ *P*<0.05, ^**^ *P*<0.01, *vs* vehicle group (control).

To better understand the role of endogenous H_2_S in the cyotoxicity of FA, the effects of FA on the cell viability and H_2_S generation in PC12 cells were explored. As shown in Fig. 1A, 24 h exposure of PC12 cells to FA (120 and 240 µmol/L) significantly inhibited cell viability, demonstrating the cytotoxicity of FA. We simultaneously evaluated whether FA alters the production of H_2_S in PC12 cells. After 24 h treatment of PC12 cells with FA (120 and 240 µmol/L), the content of H_2_S in cell medium was markedly attenuated (Fig. 1B), indicating that FA has the ability to inhibit the production of H_2_S in PC12 cells. These results imply that the inhibited H_2_S generation is involved in the cytotoxicity of FA.

### 2. Formaldehyde Inhibits H_2_S Synthesizing Activity and Down-regulates CBS Protein Expression in PC12 Cells

**Figure 2:**
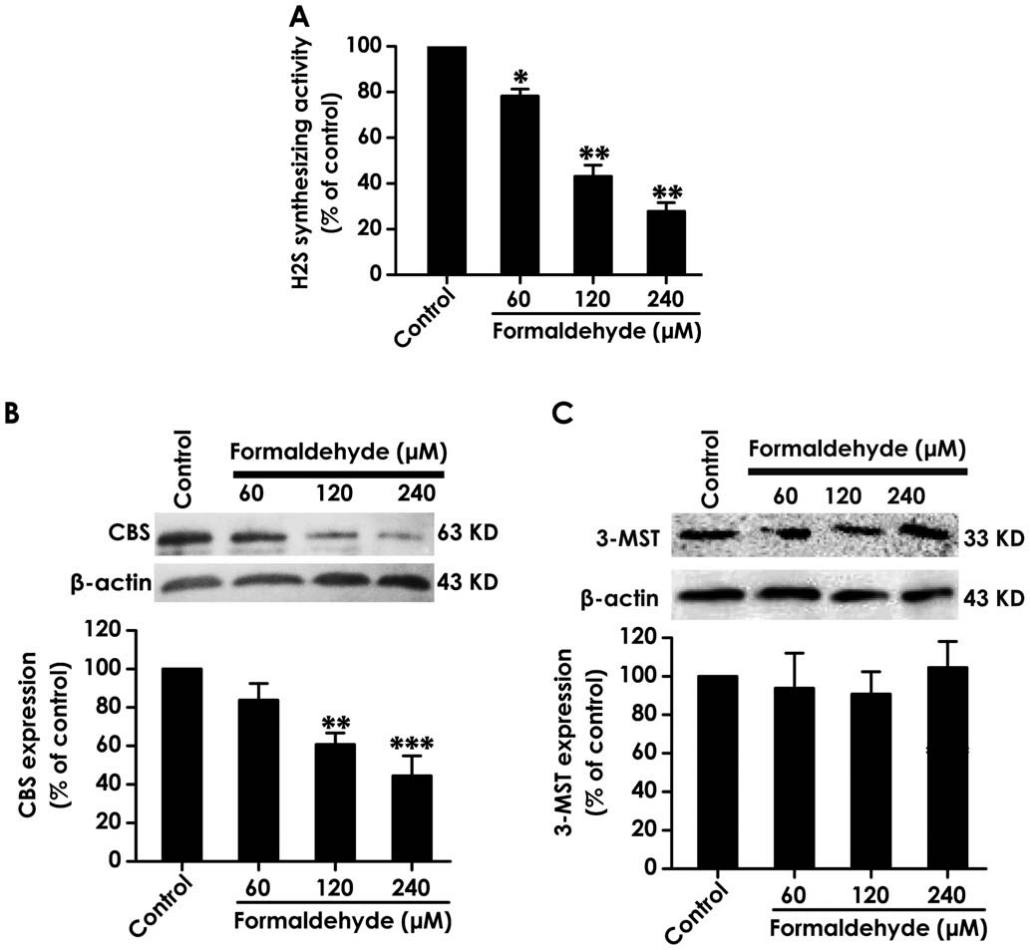
Effects of formaldehyde on H_2_S synthesizing activity and CBS and 3-MST expressions in PC12 cells. PC12 cells were treated with 60, 120, or 240 µmol/L of formaldehyde for 24 h, respectively. A, the H_2_S synthesizing activity in PC12 cells were measured by the N,Ndimethyl-p-phenylenediamine sulphate (NNDPD) method as described in “Materials and Methods” section. B, C, the levels of CBS (B) and 3-MST (C) expression in PC12 cells were determined by Western blot using anti-CBS (B) and anti-3-MST (C) antibody. Western blot images show representative results from three independent experiments. In all blots, β-actin was used as a loading control. Values are expressed as the mean ± SEM of three independent experiments. ^*^ *P*<0.05, ^**^ *P*<0.01, ^***^ *P*<0.001, *vs* vehicle group (control).

To explore whether FA-inhibited H_2_S generation is involved in down-regulation of H_2_S synthesizing activity, the effects of FA on the activity of H_2_S generation in PC12 cells were investigated. As shown in Fig.2A, the ability of H_2_S generation in PC12 cells was significantly reduced by treatment of PC12 cells with FA (60, 120 and 240 µmol/L) for 24 h, suggesting that FA-downregulated the H_2_S synthesizing activity in PC12 cells contribute to its inhibitory effect on H_2_S generation.

We have found that PC12 cells generate H_2_S by cystathionine-β-synthetase (CBS), not by cystathionine-γ-lyase (CSE) [31]. Recently, 3-mercaptopyruvate sulfur transferase (3-MST) is found as a major H_2_S producing pathway [41]. Thus, we investigated the effect of FA on the expressions of CBS and 3-MST. As shown in Fig. 2B, after 24 h exposure of FA (120 and 240 µmol/L), the expressions of CBS in PC12 cells were significantly down-regulated. However, treatment with FA (60, 120 and 240 µmol/L) for 24 h did not alter the expression of 3-MST in PC12 cells (Fig. 2C). These data suggested that FA inhibits the generation of H_2_S in PC12 cells by downregulating CBS expression, not by downregulating 3-MST expression.

### 3. CBS Silencing not only Inhibits Endogenous H_2_S Generation but also Deteriorates Formaldehyde-induced Decrease in Endogenous H_2_S Generation in PC12 Cells

**Figure 3:**
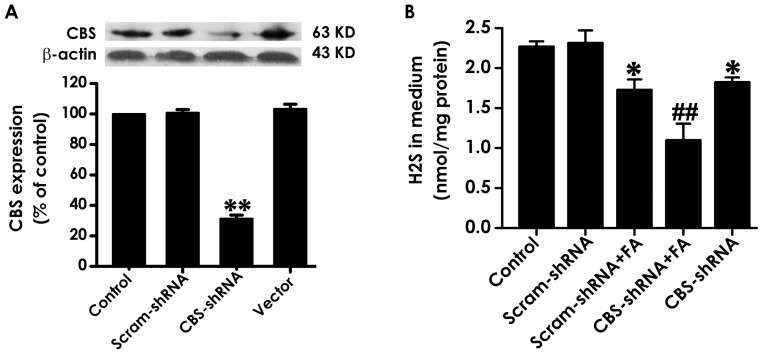
Effects of CBS-shRNA on CBS expression and endogenous H_2_S production in PC12 cells. A, PC12 cells were transfected with Scram-shRNA, CBS-shRNA, Vector for 6 h, respectively, and then the cell supernatant was sucked out and replaced with the normal cells medium. After 24 h incubation, the expression of CBS in PC12 cells was detected by Western blot using an anti-CBS antibody. Western blot images show representative results from three independent experiments. In all blots, β-actin was used as a loading control. B, after transfection with CBS-shRNA for 6 h, PC12 cells were exposed to 120 µmol/L of FA for 24 h, and then the content of H_2_S in cell medium was detected by NNDPD method as described in “Materials and Methods” section. Values are expressed as the mean ± SEM of three independent experiments. ^*^ *P*<0.05, ^**^ *P*<0.01, *vs* vehicle group (control); ^##^ *P*<0.01, *vs* FA-treated alone group.

Transfection of PC12 cells with CBS-shRNA for 6 h significantly inhibited the expression of CBS (Fig. 3A) and the generation of endogenous H_2_S (Fig. 3B). Furthermore, PC12 cells that were silenced for CBS and then exposed to FA generated significantly less H_2_S than the cells that were exposed to FA alone (Fig. 3B), indicating that knockdown of CBS deteriorates FA-inhibited endogenous H_2_S production.

### 4. Knockdown of CBS not only Induces Neurotoxicity to PC12 but also Aggravates the Neurotoxicity of Formaldehyde

**Figure 4:**
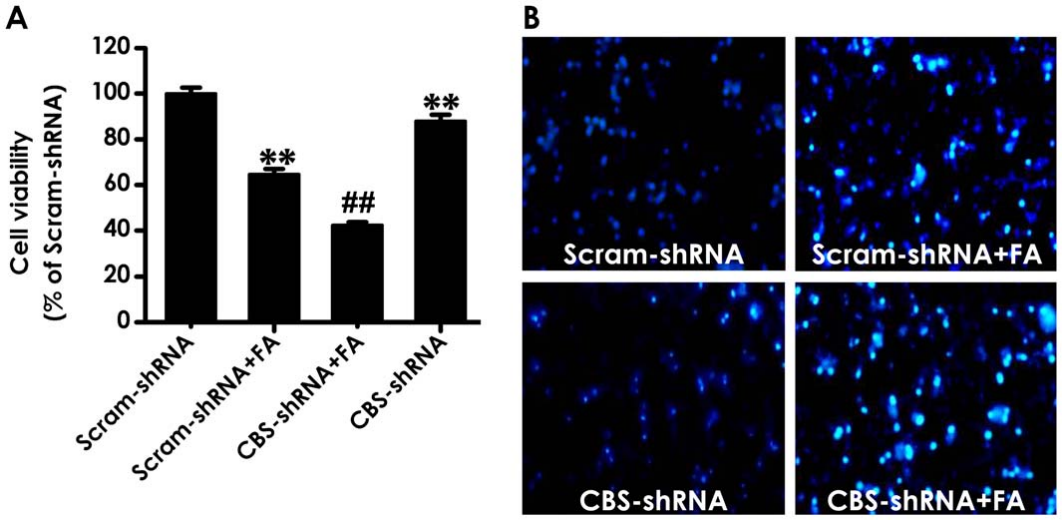
Effects of CBS-shRNA on formaldehyde-caused cytotoxicity and apoptosis in PC12 cells. After transfection with CBS-shRNA for 6 h, PC12 cells were exposed to 120 µmol/L of FA for 24 h. A, the viability of PC12 cells was determined by CCK-8 assay. B, the apoptosis of PC12 cells was visualized under a fluorescence microscope (10 × objective, BX50-FLA, Olympus) after incubated with 5 mg/L Hoechst 33258 for 30 min. Values are expressed as the mean ± SEM of three independent experiments. ^**^ *P*<0.01, *vs* scrambled shRNA group; ^##^ *P*<0.01, *vs* FA-treated alone group.

To further confirm that inhibition of endogenous H_2_S generation plays an important role in the neurotoxicity of FA, we explored the effects of CBS silencing on FA-induced cytotoxicity and apoptosis in PC12 cells. As shown in Fig. 4A, CBS-shRNA not only attenuated the viability of PC12 cells but also aggravated the decrease in the viability of PC12 cells treated with FA (120 µmol/L, 24 h).

The nuclear staining assay was used to assess the morphological changes of apoptosis in PC12 cells. As illustrated in Fig. 4B, the untreated cells exhibited uniformly dispersed chromatin and intact cell membrane. On the other hand, the FA-treated cells (120 µmol/L, 24 h) and the cells transfected with CBS-shRNA appeared typical characteristics of apoptosis, including apoptotic nuclear condensation. When PC12 cells were silenced for CBS and then exposed to FA for 24 h, however, the number of cells with nuclear condensation was significantly increased, suggesting that knockdown of CBS deteriorates FA–induced apoptosis in PC12 cells.

### 5. Knockdown of CBS Aggravates Formaldehyde-induced ROS Accumulation in PC12 Cells

**Figure 5:**
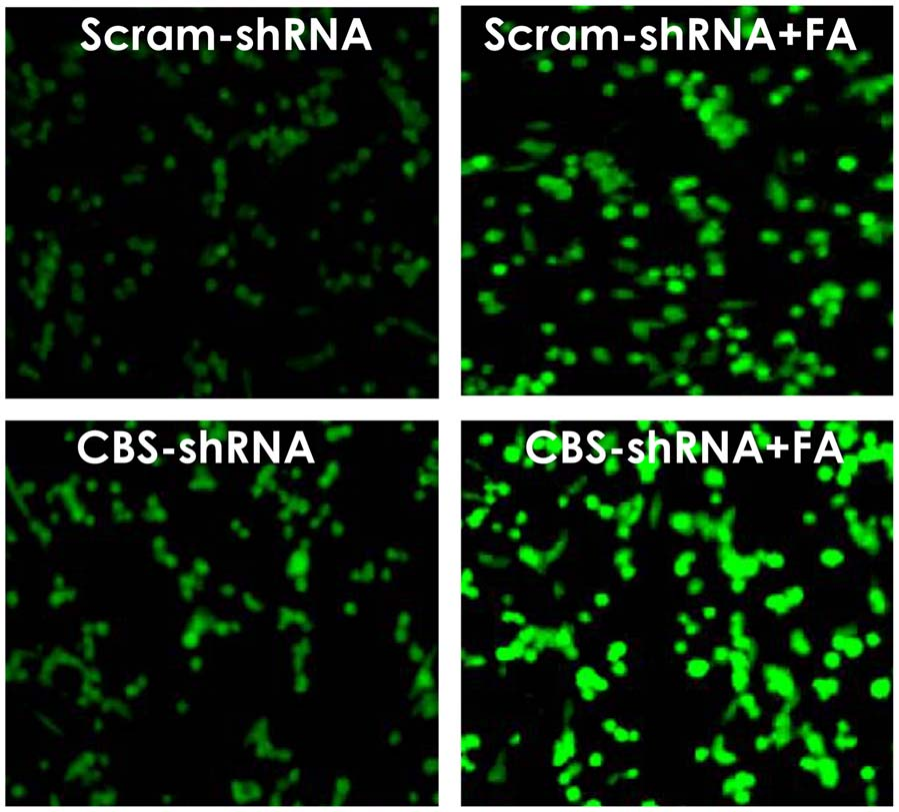
Effects of CBS-shRNA on formaldehyde-caused intracellular ROS accumulation in PC12 cells. After transfection with CBS-shRNA for 6 h, PC12 cells were exposed to 120 µmol/L of FA for 9 h and the accumulation of intracellular ROS in PC12 cells were visualized under a fluorescence microscope (10 × objective, BX50-FLA, Olympus) after DCFH-DA staining for 30 min.

Given that ROS play an important role in the neurotoxicity of FA and that H_2_S is an endogenous antioxidant gas, we wondered whether CBS silencing induces intracellular ROS accumulation and aggravates FA-induced intracellular ROS accumulation in PC12 cells. Compared with non-treated control cells, the level of intracellular ROS was increased in PC12 cells treated with 120 µmol/L of FA for 9 h or transfected with CBS-shRNA, as shown by the increase in DCF fluorescence (Fig. 5). However, when PC12 cells were silenced for CBS and then exposed to FA for 9 h, the DCF fluorescence were significantly increased (Fig. 5), suggesting that knockdown of CBS deteriorates FA–induced intracellular ROS accumulation in PC12 cells.

### 6. Formaldehyde Increases the Levels of nNOS and iNOS as well as the Generation of NO in PC12 Cells

**Figure 6:**
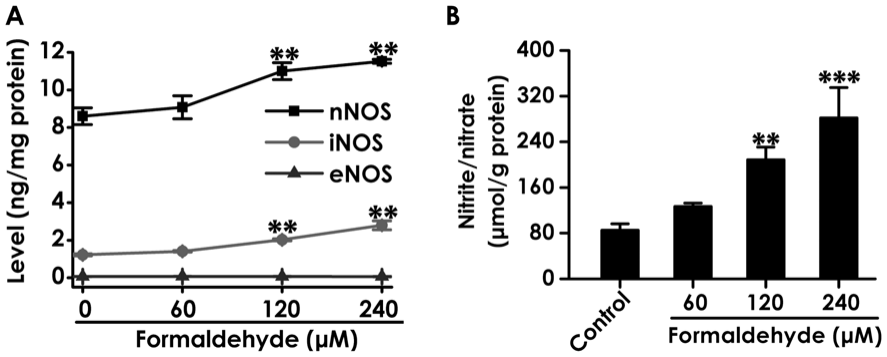
Effects of formaldehyde on the levels of NOS isoforms and the generation of NO in PC12 cells. After treatment of PC12 cells with 60, 120, and 240 µmol/L of FA for 24 h, respectively, the levels of NOS isoforms in PC12 cells (A) and the content of NO in cell medium (B) were determined by ELISA kit and NO kit, respectively. Values are expressed as the mean ± SEM of three independent experiments. ^**^ *P*<0.01, ^***^ *P*<0.001, *vs* vehicle group (control).

It has been proved that NO binds to CBS and overproduction of NO inhibits the activity of CBS [37], [38]. To gain insight into whether the mechanism of FA-induced inhibition in endogenous H_2_S generation is involved in overproduction of NO, the effects of FA on the levels of NOS isoforms and the generation of NO in PC12 cells were explored. The level of eNOS in PC12 cells was not measured. After 24 h exposure of FA (120 and 240 µmol/L), the levels of nNOS and iNOS (Fig. 6A) and the production of NO (Fig. 6B) in PC12 cells were significantly increased. These results suggested that FA-inhibited endogenous H_2_S generation may be associated with its role in upregulation of NOS and overproduction of NO.

### 7. Asymmetric Dimethylarginine (ADMA), an Endogenous NOS Inhibitor, Attenuates Formaldehyde-induced Excess of NOS Level and NO Production

**Figure 7:**
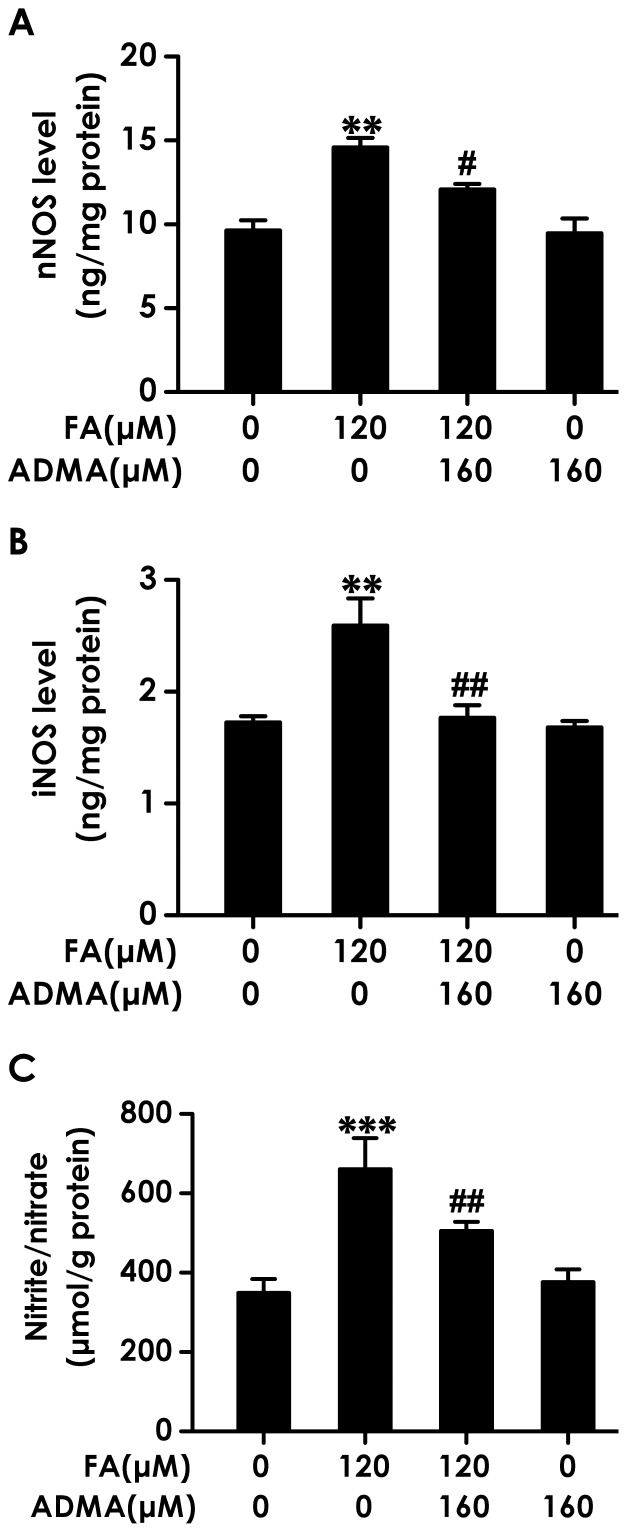
Effects of ADMA on formaldehyde-induced increases in the levels of NOS isoforms and the generation of NO in PC12 cells. After pretreated with 160 µmol/L of ADMA for 30 min, PC12 cells were exposed to formaldehyde (FA, 120 µmol/L) for 24 h. The levels of nNOS (A) and iNOS (B) and the generation of NO (C) in PC12 cells were measured by ELISA kits and NOS kits, respectively. Values are expressed as the mean ± SEM of three independent experiments. ^**^ *P*<0.01, ^***^ *P*<0.001, *vs* vehicle control group; ^#^ *P*<0.05, ^##^ *P*<0.01, *vs* FA-treated alone group.

ADMA is an endogenous NOS inhibitor. Firstly, we aimed to explore whether ADMA attenuates FA-induced excess upregulation of NOS and overproduction of NO. Pretreatment of PC12 cells with 160 µmol/L of ADMA for 30 min markedly reversed FA (120 µmol/L, for 24 h)-induced increase in the levels of nNOS (Fig. 7A) and iNOS (Fig. 7B) and the generation of NO (Fig. 7C). These data indicated that ADMA can prevent FA-induced overproduction of NO.

### 8. ADMA Reverses the Inhibitions of CBS Expression as well as the Decrease of H_2_S Generation Induced by Formaldehyde

**Figure 8:**
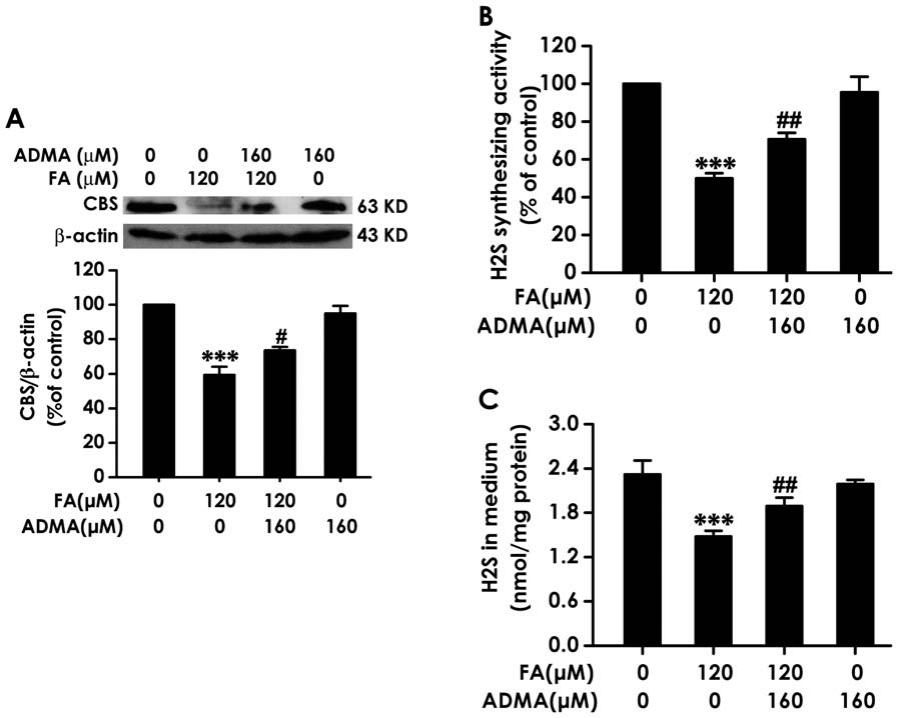
Effects of ADMA on formaldehyde-induced inhibition of CBS expression as well as decrease in H_2_S generation in PC12 cells. After pretreated with 160 µmol/L of ADMA for 30 min, PC12 cells were exposed to formaldehyde (FA, 120 µmol/L) for 24 h. A, The levels of CBS expression in PC12 cells were determined by Western blot using an anti-CBS antibody. Western blot images show representative results from three independent experiments. In all blots, β-actin was used as a loading control. B, The H_2_S synthesizing activity in PC12 cells were measured by the NNDPD method as described in “Materials and Methods” section. C, The content of H_2_S in cell culture supernatant was tested by the NNDPD method as described in “Materials and Methods” section. Values are expressed as the mean ± SEM of three independent experiments. ^***^ *P*<0.001, *vs* vehicle control group; ^#^ *P*<0.05, ^##^ *P*<0.01, *vs* FA-treated alone group.

We next assessed whether ADMA reverses FA-induced inhibition in the activity and expression of CBS and the generation of endogenous H_2_S in PC12 cells. Pretreatment of PC12 cells with 160 µmol/L of ADMA for 30 min markedly attenuated FA (120 µmol/L, for 24 h)-induced decrease in the expression of CBS (Fig. 8A), the activity of H_2_S generation (Fig. 8B) in PC12 cells and the level of H_2_S in cell medium (Fig. 8C), which suggested that FA inhibits endogenous H_2_S generation by overproduction of NO.

### 9. ADMA Prevents Formaldehyde-induced Cytotoxicity and Apoptosis in PC12 Cells

**Figure 9:**
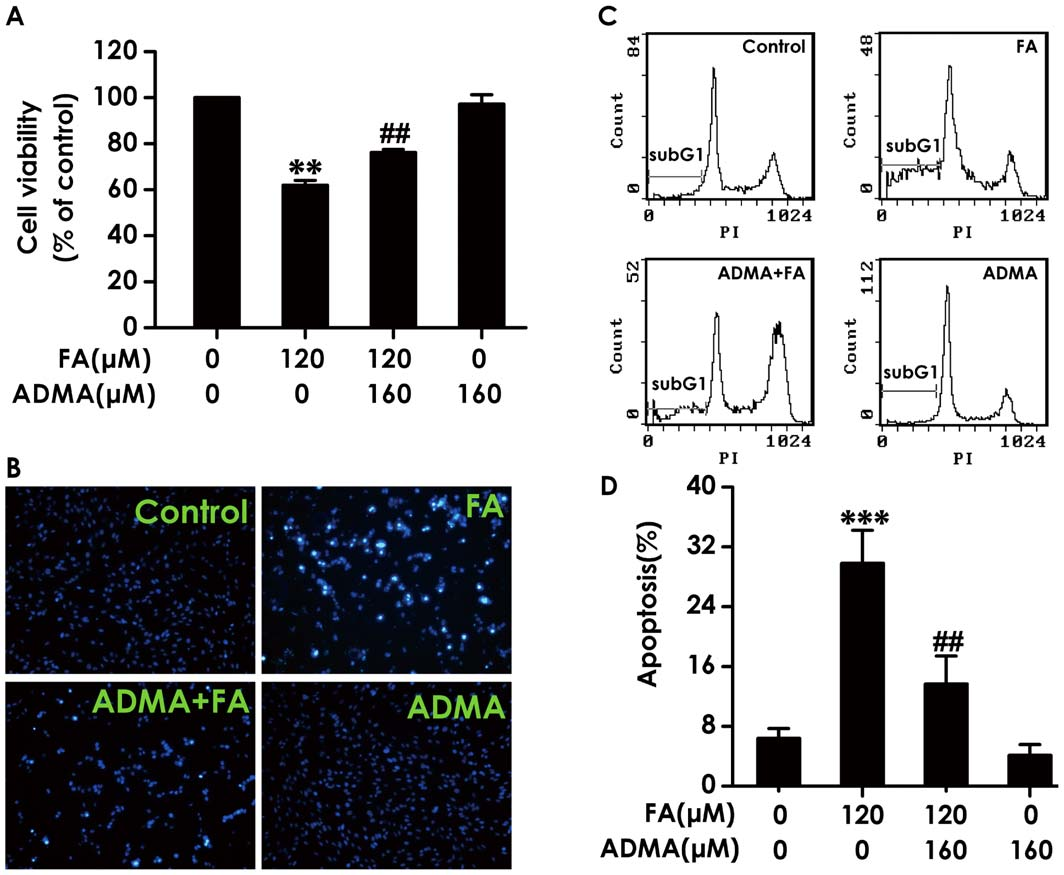
Effects of ADMA on formaldehyde-induced cytotoxicity and apoptosis in PC12 cells. After pretreated with 160 µmol/L of ADMA for 30 min, PC12 cells were exposed to formaldehyde (FA, 120 µmol/L) for 24 h. A, The cell viability was determined by CCK-8 assay. B, The apoptosis of PC12 cells was visualized under a fluorescence microscope (10 × objective, BX50-FLA, Olympus) after incubated with 5 mg/L Hoechst 33258 for 30 min. C, D, The apoptosis of PC12 cells was assessed by flow cytometry after PI staining. (C) Representative DNA histogram of PC12 cells exposed to different treatments. In the DNA histogram, the amplitude of the sub-G1 DNA peak represents the number of apoptotic cells. (D) Quantitative analysis of the rate of apoptosis. Values are expressed as the mean ± SEM of three independent experiments. ^**^ *P*<0.01, ^***^ *P*<0.001, *vs* vehicle control group; ^##^ *P*<0.01, *vs* FA-treated alone group.

Next, to determine whether ADMA reverses the neurotoxicity of FA, the effects of ADMA on FA-elicited cytotoxicity and apoptosis in PC12 cells were examined. As shown in Fig. 9A, the cytotoxic effect of FA (120 µmol/L, for 24 h) on PC12 cells was significantly blocked by pretreatment with 160 µmol/L of ADMA for 30 min.

The beneficial effects of ADMA against the neurotoxicity of FA were further examined by apoptosis assay. The nuclear staining assay was used to assess the morphological changes of apoptosis in PC12 cells. When PC12 cells were pretreated with 160 µmol/L of ADMA for 30 min, the number of cells with typical characteristics of apoptosis induced by FA (120 µmol/L, 24 h) was significantly attenuated (Fig. 9B), suggesting that ADMA protects PC12 cells against FA-induced apoptosis.

The finding from FCM analysis after PI staining for apoptosis also indicated that ADMA protects PC12 cells against apoptosis induced by FA. As shown in Fig. 9C, exposure of PC12 cells to FA (120 µmol/L, 24 h) caused significant apoptosis and the apoptotic effects induced by FA were prevented by pretreatment with ADMA (160 µmol/L, 30 min).

### 10. ADMA Prevents Formaldehyde-induced ROS Accumulation in PC12 Cells

**Figure 10:**
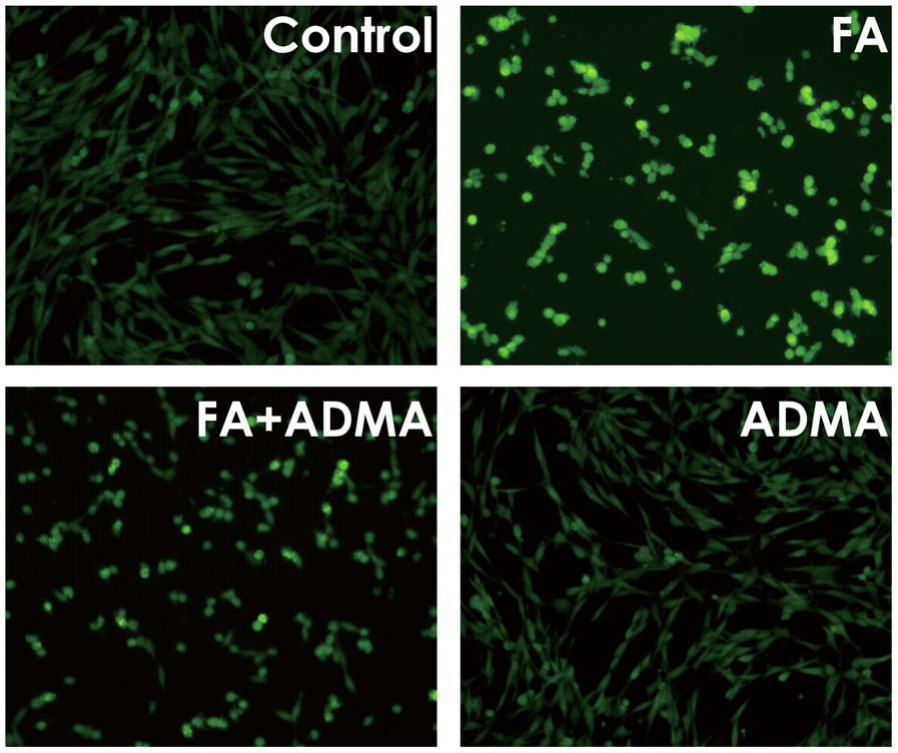
Effects of ADMA on formaldehyde-caused intracellular ROS accumulation in PC12 cells. After pretreated with 160 µmol/L ADMA for 30 min, PC12 cells were exposed to formaldehyde (FA, 120 µmol/L) for 9 h and the accumulation of intracellular ROS in PC12 cells were visualized under a fluorescence microscope (10×objective, BX50-FLA, Olympus) after DCFH-DA staining for 30 min.

Given that ROS plays an important role in the neurotoxicity of FA, we wondered whether ADMA prevents FA-induced intracellular ROS accumulation in PC12 cells. Compared with non-treated control cells, the level of intracellular ROS was increased in PC12 cells treated with 120 µmol/L of FA for 9 h, as shown by the increase in DCF fluorescence (Fig. 10). However, when PC12 cells were pretreated with ADMA (160 µmol/L, 30 min), the DCF fluorescence was significantly decreased (Fig. 10). These results suggested that ADMA reverses FA-induced intracellular ROS accumulation.

## Discussion

The present study shows that treatment of PC12 cells with FA leads to (i) cytotoxicity; (ii) inhibition of CBS expression and decrease in endogenous H_2_S production; and (iii) increase in the levels of nNOS and iNOS and overproduction of NO. Knockdown of CBS using shRNA deteriorates FA-induced decreases in endogenous H_2_S generation, neurotoxicity, and intracellular ROS accumulation in PC12 cells. We also show that inhibition of NOS by its specific inhibitor (ADMA) significantly attenuates FA-induced decreases in endogenous H_2_S generation, neurotoxicity, and intracellular ROS accumulation in PC12 cells. Collectively, these findings suggest that FA confers neurotoxicity by inhibiting the generation of H_2_S through overproduction of NO.

Our previous findings that treatment of PC12 cells with FA reduces the viability of cell and promotes the release of LDH demonstrate the neurotoxicity of FA. It has been shown that FA negatively affects learning and memory in animals [10], [42] and siginficantly leads to neuronal damage in the prefrontal cortex of rats including the hippocampus [8], [9]. Song and colleagues reported that exposure of the cultured cortical neurons to FA causes neurotoxic effects [4]. These previous results provide strong evidence to support the neurotoxicity of FA. However, the mechanisms involved in FA toxicity to neural cells have not been fully clarified.

H_2_S, characterized with an odor of rotten eggs, is a poisonous gas used as chemical reagent. Nevertheless, more and more literatures reported that H_2_S involves in many physiological functions, especially in the nervous system and cardiovascular system [43], [44]. H_2_S has been referred to as the third gaseous signaling molecule alongside NO and carbon monoxide (CO) [43], [45], [46]. In the present study, we showed that FA decreases the generation of H_2_S in PC12 cells. CBS is the major enzyme responsible for endogenous H_2_S generation in PC12 cells [31]. In this study, we also demonstrated the downregulation of CBS expression in FA-treated PC12 cells. These data indicated that FA disturbs the generation of endogenous H_2_S in PC12 cells by inhibition of CBS expression. It has been reported that oxidative damage is one of the most critical effects of FA exposure [18]. Lu and colleagues have demonstrated that FA causes deficit in learning and memory by oxidative stress [10]. Our previous results have demonstrated that PC12 cells exposed to FA has a significant increase in intracellular ROS accumulation and indicated that FA-caused oxidative stress plays a critical role in its neurotoxicity [5]. Oxidative stress occurs from either a overproduction of ROS or a decline in antioxidant defenses [47]. Unbalanced in proportion to protective antioxidants causes the excess ROS and ultimately leads to cell death [20], [21]. H_2_S is an endogenous antioxidant gas [22]. Thus, we hypothesize that FA-inhibited H_2_S generation is involved in its neurotoxicity.

To confirm whether the disturbance of H_2_S synthesis is a novel mechanism underlying FA-induced neurotoxicity, we investigated the effects of inhibited H_2_S generation by CBS knockdown on the neurotoxicity of FA. In this present work, we showed that inhibition of endogenous H_2_S production by CBS silencing using CBS siRNA aggravates FA-induced cytotoxicity, apoptosis, and accumulation of intracellular ROS in PC12 cells. Our previous results that treatment with NaHS, a H_2_S donor, suppresses FA-induced cytotoxicity, apoptosis, loss of mitochondrial membrane potential, and accumulation of intracellular ROS have demonstrated that exogenous application of H_2_S reverses the neurotoxicity and oxidative stress induced by FA [48]. Taken together, our previous and present findings indicate that FA-disturbed H_2_S production plays an important role in its neurotoxicity. Disturbed H_2_S synthesis has been involved in Down’s syndrome [49], stroke [50] and possibly Alzheimer’s disease [51]–[53]. We have previously demonstrated that inhibition of H_2_S production contributes to the neurotoxicity induced by MPP^+^ and homocystene [31], [32]. Those previous findings provide reasonable explanation for our results. Thus, we suggest that FA-induced inhibition of CBS expression and decrease in endogenous H_2_S generation results in deficiency in ROS scavenging, which in turn leads to ROS accumulation and ultimately causes neurotoxic effect.

In the present study, we further examined the mechanism of FA-induced disturbance of H_2_S synthesis. It has been reported that NO is able to inhibit CBS activity [37], [38]. Recently, the result from Songur demonstrated that inhalation of FA significantly increases the level of NO in the cerebellar tissue [36]. It is logical for us to assume that overproduction of NO is responsible for the role of FA in the disturbance of H_2_S synthesis in PC12 cells. In the present work, we showed that treatment of PC12 with FA leads to excess of NO production, which is consistent with the result from Songur [36]. Since the expression of CBS and the activity of H_2_S generation in PC12 cells were significantly suppressed by treatment with FA, we hypothesized that the elevated NO level during FA treatment might play roles in downregulating the expression of CBS leading to decrease in H_2_S generation. Levels of NO remained elevated in PC12 cells during FA treatment periods, while a significant increase in the levels of nNOS and iNOS was also detected in the FA-exposed PC12 cells. To confirm whether the elevation of NO production mediates the decrease of H_2_S synthesis, we investigated the effects of ADMA, a specifical NOS inhibitor, on FA-induced elevation of nNOS and iNOS levels and NO generation as well as inhibition of endogenous H_2_S production in PC12 cells. Our present data showed that pretreatment with ADMA not only reverses FA-induced increase in the levels of nNOS and iNOS and the production of NO but also recovers the expression of CBS and the endogenous generation of H_2_S. Therefore, these findings revealed that FA-induced NO overproduction contributes to its roles in inhibiting the expression of CBS and the generation of H_2_S in PC12 cells. NO and H_2_S are a growing family of regulatory gaseous molecules involved in regulation of physiological and pathological functions in mammalian tissues. They can not only exert comparable biological actions but also compete with and are antagonists with each other [54]. Recently, it has been demonstrated that H_2_S directly inhibits the generation of NO [55]. Our data provide a novel aspect for understanding of the interaction between two gasotransmitters, H_2_S and NO.

Finally, we explored whether inhibition of FA-induced NO overproduction by ADMA could ameliorate the neurotoxicity and oxidative stress induced by FA. The present results demonstrated that pretreatment with ADMA to inhibit FA-elicited NO overproduction prevents FA-caused cytotoxicity, apoptosis, and accumulation of ROS in PC12 cells. These findings further support the notion that NO, at elevated levels, contributes to inhibition of CBS activity and decrease in H_2_S generation, which in turn results in ROS accumulation and ultimately leads to neurotoxic effect.

In conclusion, the present work showed that FA inhibits the expression of CBS and the generation of H_2_S as well as elevates the production of NO. Inhibition of H_2_S generation by knockdown of CBS aggravated the neurotoxicity of FA, while suppressed NO overproduction by ADMA, a specifical NOS inhibitor, not only rescued FA-induced downregulation of CBS and decrease in H_2_S generation but also prevented the neurotoxicity of FA. These results clearly identify that the neurotoxicity of FA is contributed to the inhibited endogenous H_2_S production, which is mediated by overproduction of NO. The present findings offer a novel mechanistic explanation to the neurotoxicity of FA. Based on the current findings, we propose that novel therapeutic approaches for FA neurotoxicity should focus on the restoration of NO and H_2_S homeostasis in neurons.
